# Supplementary material for: Inhibition of Quorum-Sensing Regulator from Pseudomonas aeruginosa Using a Flavone Derivative
Source: Molecules. 2022 Apr 10;27(8):2439. doi: 10.3390/molecules27082439 (PMC9031925; doi:10.3390/molecules27082439)
Supplement: Supplementary file 1 [file molecules-27-02439-s001.zip › molecules-1631139-supplementary.pdf]

Supplementary Materials:

Inhibition of Quorum-Sensing Regulator from *Pseudomonas aeruginosa* Using a Flavone Derivative

Yanxuan Xie [1,2], Jingxin Chen [1,2], Bo Wang [1], Ai-Yun Peng [1], Zong-Wan Mao [1,2,*] and Wei Xia [1,2,*]

1 School of Chemistry, Sun Yat-Sen University, Guangzhou 510275, China; xieyx8@mail3.sysu.edu.cn (Y.X.); chenjx256@mail3.sysu.edu.cn (J.C.); ceswb@mail.sysu.edu.cn (B.W.); cespay@mail.sysu.edu.cn (A.-Y.P.)

2 MOE Key Laboratory of Bioinorganic and Synthetic Chemistry, Sun Yat-Sen University, Guangzhou 510275, China

* Correspondence: cesmzw@mail.sysu.edu.cn (Z.-W.M.); xiawei5@mail.sysu.edu.cn (W.X.)

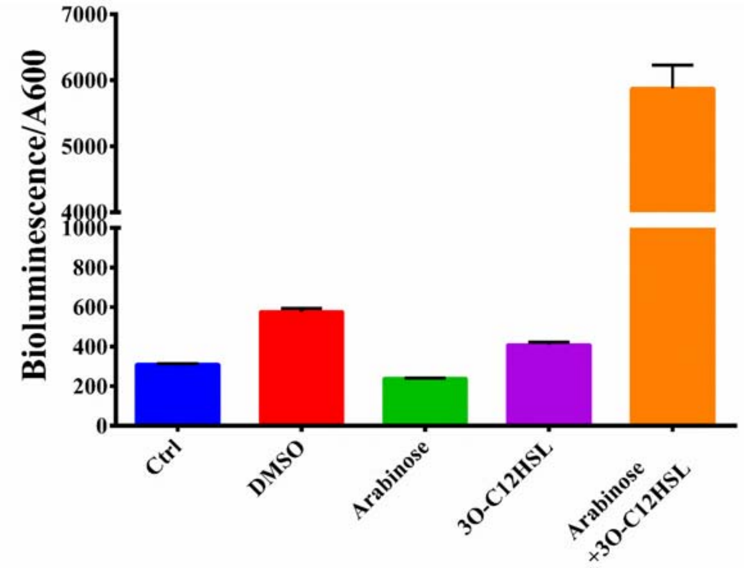

Figure S1. **The bioluminescence produced by *E. coli* reporter strain incubated with different molecules.** In the presence of 1% (v/v) DMSO, 0.1% (w/v) arabinose, 10 μM 3O-C12HSL and 0.1% (w/v) arabinose with 10 μM 3O-C12HSL. The reporter strain produces high levels of bioluminescence signals in the presence of both arabinose and 3O-C12HSL auto-inducer.

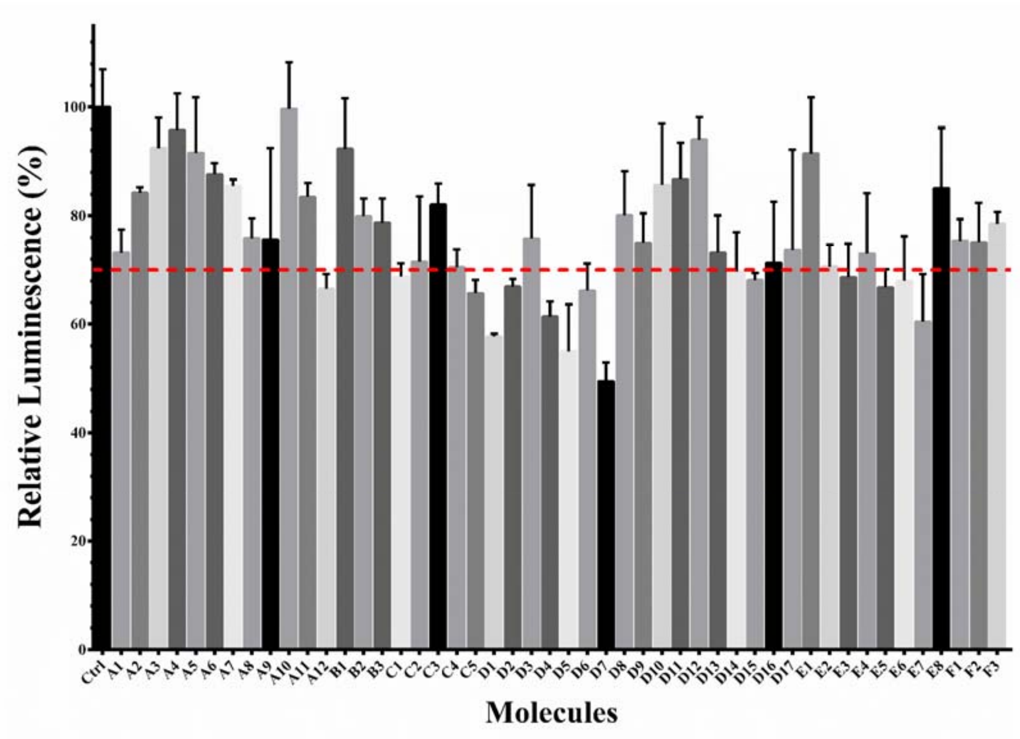

Figure S2. Screening of potential inhibitors of *P. aeruginosa las* system by the LasR *E. coli* reporter strain. All self-synthesized flavonoid and xanthone derivatives were tested at 100 µM for inhibition in the LasR *E. coli* reporter strain in the presence of 10 µM 3O-C12HSL and 0.1% (w/v) arabinose. The bioluminescence signal in the control group was set as 100%. The signal in other experimental groups were normalized to that in the control group. All experiments were performed in duplicate.

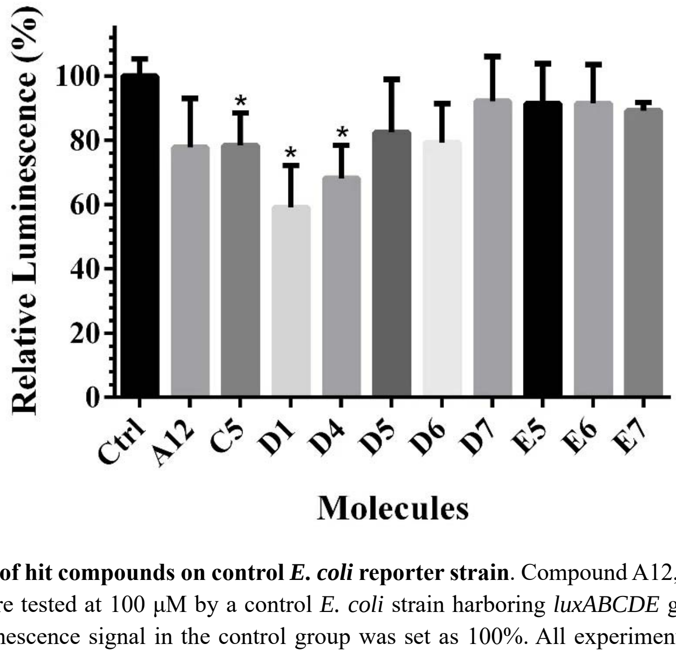

Figure S3. The effects of hit compounds on control *E. coli* reporter strain. Compound A12, C5, D1, D4, D5, D6, D7, E5, E6 and E7 were tested at 100 µM by a control *E. coli* strain harboring *luxABCDE* genes fused to the *tac* promoter. The bioluminescence signal in the control group was set as 100%. All experiments were performed in triplicate. Results are shown as mean ± sd. * $p<0.05$.

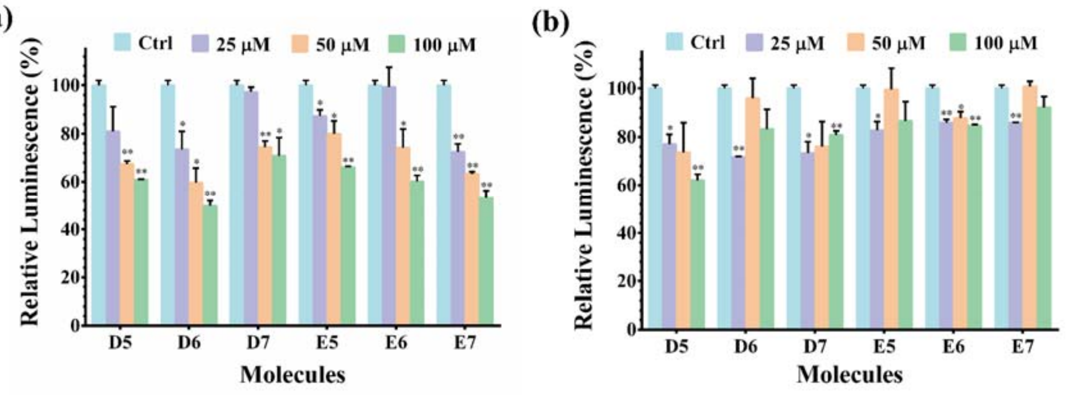

Figure S4. The effects of identified compounds on *E. coli* reporter strain bioluminescence production. (a) The bioluminescence signal of *E. coli* reporter strain was suppressed by gradient concentrations of identified compounds (25 to 100 μM). (b) The effects of identified compounds on the bioluminescence signal produced by a control *E. coli* strain harboring *luxABCDE* genes fused to *tac* promoter. All experiments were performed in triplicate. Results are shown as mean ± sd. * $p<0.05$, ** $p<0.01$.

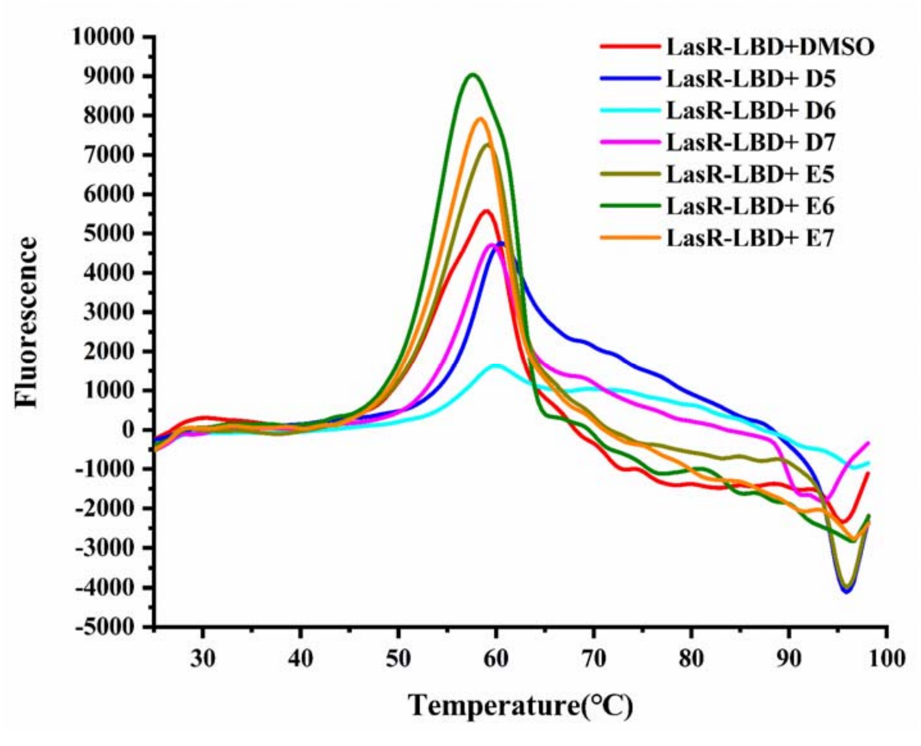

Figure S5. Fluorescent thermal shift assay of the ligand binding domain of LasR (LasR-LBD). The fluorescent thermal denaturation curves of LasR-LBD were recorded in the presence of 200 μM identified compounds as indicated.

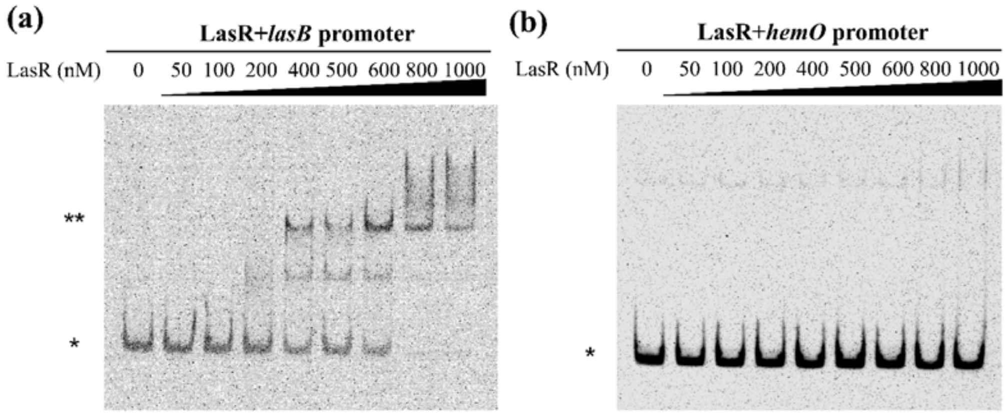

Figure S6. Electrophoretic mobility shift assay (EMSA) analysis of the LasR binding to the *lasB* and *hemO* DNA promoters. 30 ng *lasB* (a) or *hemO* (b) promoter DNA binding to gradient concentrations of full-length LasR. * indicates free DNA band, ** indicates protein-DNA complex band.

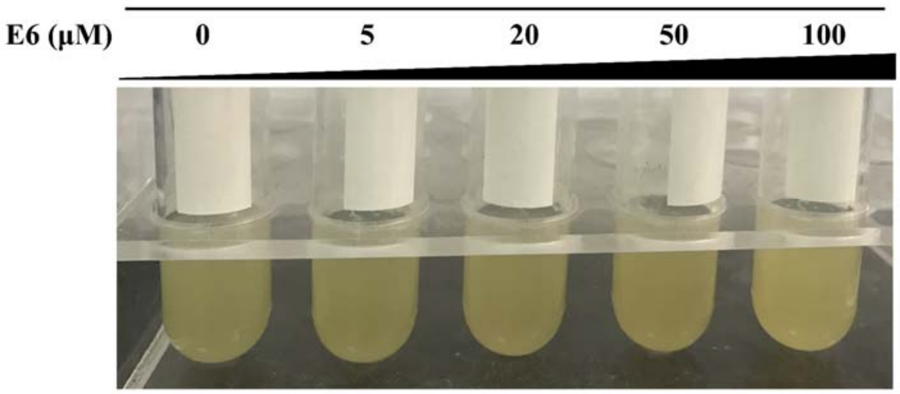

Figure S7. The effect of compound E6 on pyocyanin production of *P. aeruginosa*. The production of *P. aeruginosa* pigment pyocyanin gradually decreased with the concentration of compound E6 increased from 0 to 100 μM.

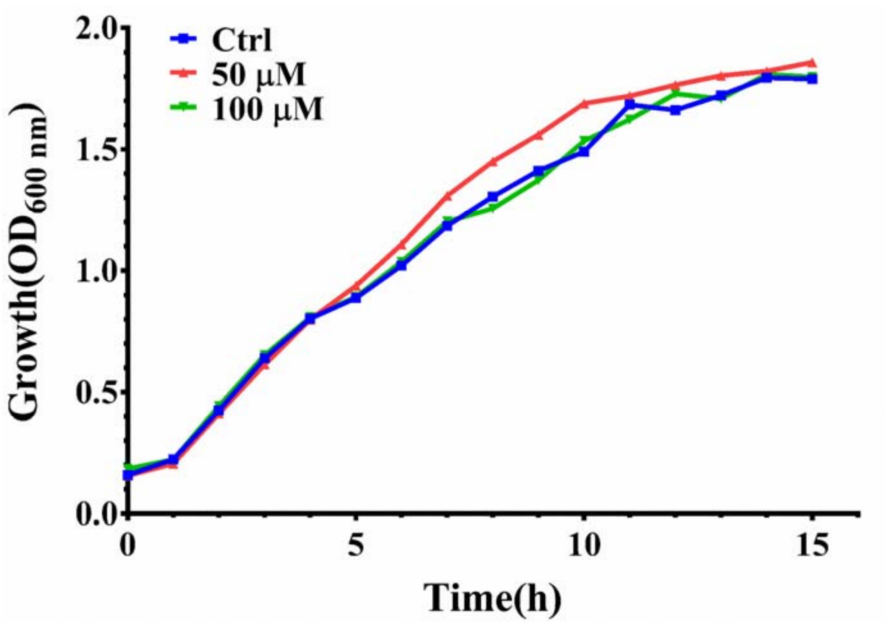

Figure S8. The effects of compound E6 on the growth of *P. aeruginosa*. Different concentrations of compound E6 were added in *P. aeruginosa* bacterial culture when OD_{600} reached 0.1. The OD_{600} was recorded at 60 min intervals.

Table S1. The structure of self-synthesized compounds * [1–5].

Number	Compound	Structure
A1	1,3-dihydroxy-9H-xanthen-9-one	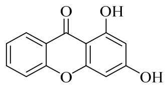
A2	1,3,6-trihydroxy-9H-xanthen-9-one	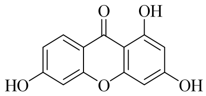
A3	1,3,7-trihydroxy-9H-xanthen-9-one	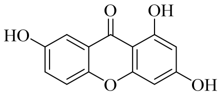
A4	9-oxo-9H-xanthene-1,3-diyl diacetate	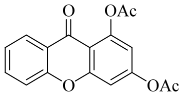
A5	9-oxo-9H-xanthen-1-yl acetate	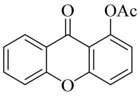
A6	9-oxo-9H-xanthene-1,6-diyl diacetate	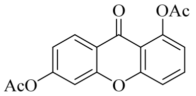
A7	9-oxo-9H-xanthene-1,3,7-triyl triacetate	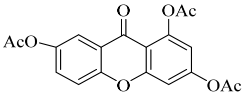
A8	1-hydroxy-3-(pentyloxy)-9H-xanthen-9-one	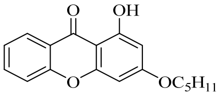
A9	1-hydroxy-3-(octyloxy)-9H-xanthen-9-one	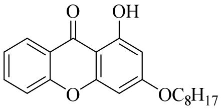
A10	(1,8-dihydroxy-9-oxo-9H-xanthene-3,6-diyl)bis(oxiran-2-ylmethanone)	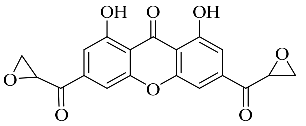
A11	1-hydroxy-3-(3-(piperidin-1-yl)propanoyl)-9H-xanthen-9-one	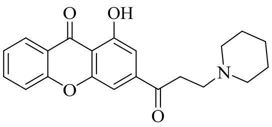
A12	1-hydroxy-3-(2-hydroxyethoxy)-9H-xanthen-9-one	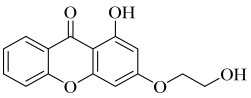

B1	1,3-dihydroxy-12H-benzo[b]xanthen-12-one	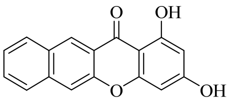
B2	1,3,9-trihydroxy-12H-benzo[b]xanthen-12-one	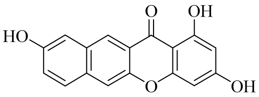
B3	1,3,7-trihydroxy-12H-benzo[b]xanthen-12-one	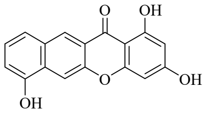
C1	1-hydroxy-9-oxo-9H-xanthen-3-yl cinnamate	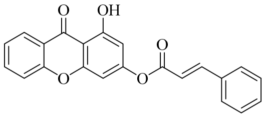
C2	1-hydroxy-9-oxo-9H-xanthen-3-yl 4-methylbenzoate	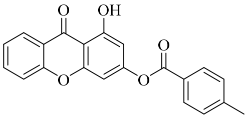
C3	1-hydroxy-9-oxo-9H-xanthen-3-yl 4-methoxybenzoate	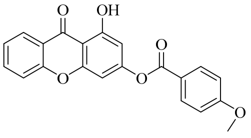
C4	1-hydroxy-9-oxo-9H-xanthen-3-yl 4-((tert-butyldimethylsilyl)oxy)benzoate	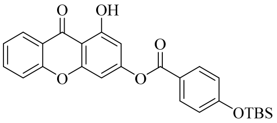
C5	1-hydroxy-9-oxo-9H-xanthen-3-yl 2-chlorobenzoate	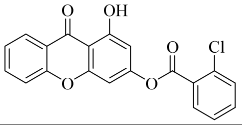
D1	N-(6,8-dihydroxy-9-oxo-9H-xanthen-3-yl)-4-methoxybenzenesulfonamide	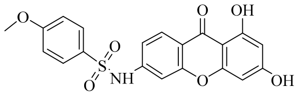
D2	N-(6,8-dihydroxy-9-oxo-9H-xanthen-3-yl)-4-ethoxybenzenesulfonamide	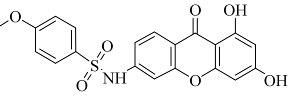
D3	N-(4-(N-(6,8-dihydroxy-9-oxo-9H-xanthen-3-yl)sulfamoyl)phenyl)acetamide	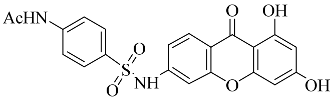
D4	N-(6,8-dihydroxy-9-oxo-9H-xanthen-3-yl)-4-hydroxybenzenesulfonamide	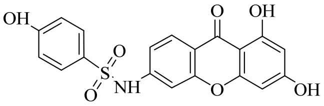
D5	4-amino-N-(6,8-dihydroxy-9-oxo-9H-xanthen-3-yl)benzenesulfonamide	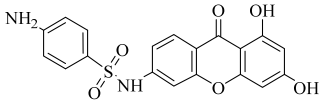
D6	4-chloro-N-(6,8-dihydroxy-9-oxo-9H-xanthen-3-yl)benzenesulfonamide	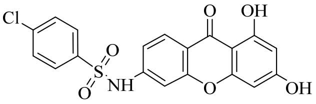

D7	N-(6,8-dihydroxy-9-oxo-9H-xanthen-3-yl)-4-fluorobenzenesulfonamide	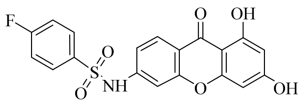
D8	N-(6,8-dihydroxy-9-oxo-9H-xanthen-2-yl)-4-methylbenzenesulfonamide	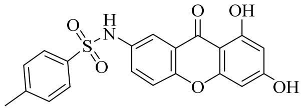
D9	N-(6,8-dihydroxy-9-oxo-9H-xanthen-2-yl)benzenesulfonamide	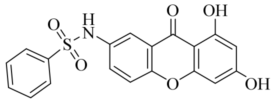
D10	N-(6,8-dihydroxy-9-oxo-9H-xanthen-2-yl)-4-methoxybenzenesulfonamide	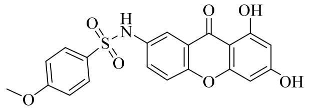
D11	N-(6,8-dihydroxy-9-oxo-9H-xanthen-2-yl)-4-ethoxybenzenesulfonamide	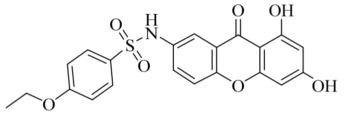
D12	4-bromo-N-(6,8-dihydroxy-9-oxo-9H-xanthen-2-yl)benzenesulfonamide	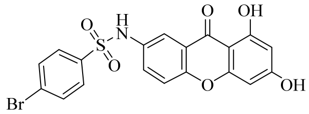
D13	N-(4-(N-(6,8-dihydroxy-9-oxo-9H-xanthen-2-yl)sulfamoyl)phenyl)acetamide	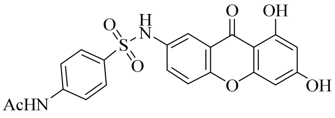
D14	N-(6,8-dihydroxy-9-oxo-9H-xanthen-2-yl)-4-hydroxybenzenesulfonamide	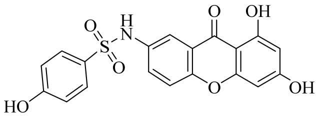
D15	4-amino-N-(6,8-dihydroxy-9-oxo-9H-xanthen-2-yl)benzenesulfonamide	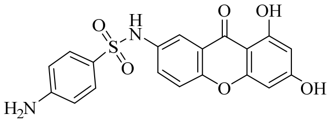
D16	4-chloro-N-(6,8-dihydroxy-9-oxo-9H-xanthen-2-yl)benzenesulfonamide	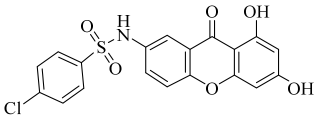
D17	N-(6,8-dihydroxy-9-oxo-9H-xanthen-2-yl)-4-fluorobenzenesulfonamide	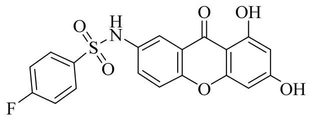
E1	7-(4-aminobutoxy)-5-hydroxy-2-phenyl-4H-chromen-4-one	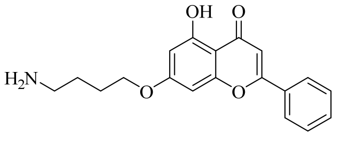
E2	5-hydroxy-7-(3-(4-(4-methoxycyclohexyl)-1H-1,2,3-triazol-1-yl)propoxy)-2-phenyl-4H-chromen-4-one	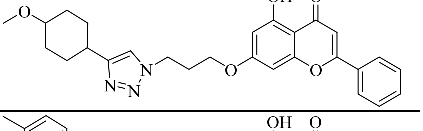
E3	5-hydroxy-2-phenyl-7-(4-(4-(p-tolyl)-1H-1,2,3-triazol-1-yl)butoxy)-4H-chromen-4-one	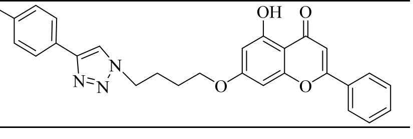

E4	7-(4-(4-(4-fluorophenyl)-1H-1,2,3-triazol-1-yl)butoxy)-5-hydroxy-2-phenyl-4H-chromen-4-one	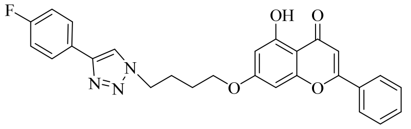
E5	diethyl (3-((5-hydroxy-4-oxo-2-phenyl-4H-chromen-7-yl)oxy)propyl)phosphoramidate	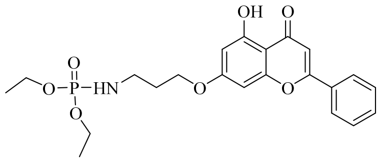
E6	dimethyl (4-((5-hydroxy-4-oxo-2-phenyl-4H-chromen-7-yl)oxy)butyl)phosphoramidate	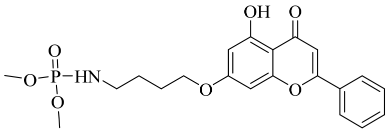
E7	diisopropyl (4-((5-hydroxy-4-oxo-2-phenyl-4H-chromen-7-yl)oxy)butyl)phosphoramidate	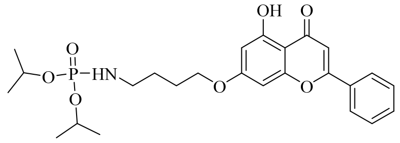
E8	diethyl (4-((4-oxo-2-phenyl-4H-chromen-7-yl)oxy)butyl)phosphoramidate	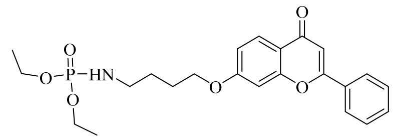
F1	4-Allyl-1-ethoxy-3-(p-tolyl)benzo[c][1,2]oxaphosphinine 1-Oxide	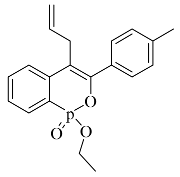
F2	4-Allyl-3-(4-methoxyphenyl)benzo[c][1,2]oxaphosphinine 1-Oxide	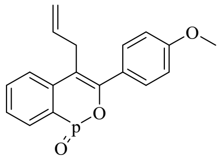
F3	4-Allyl-3-(4-fluorophenyl)benzo[c][1,2]oxaphosphinine 1-Oxide	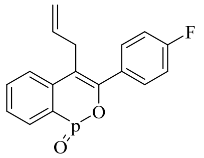

* Compound A1-A12 from reference 43, B1-B3 from reference 44, C1-C5 from reference 45, E1-E8 from reference 46, F1-F3 from reference 47, D1-D17 from "Xanthone Sulfonamide Derivatives-A Novel Series of α-Glucosidase Inhibitors with Different Inhibitory Types", which has been submitted.

Table S2. The cytotoxicity of compound E6 toward mammalian cell.

	IC_{50}			
**Cells**	**LO2**	**BEAS-2B**	**WI-38**	**A549**
**$Ga(NO_3)_3$**	>200 μM	>200 μM	>200 μM	>200 μM

**E6**	>200 μM	>200 μM	178 μM	260 μM

Table S3. The primers used in PCR amplification and qPCR.

Primer	Sequence (5′-3′)	Primer	Sequence (5′-3′)
LasR-For	AAAGAATTCAGGAGGTAAATG GCCTTGGTTGAC	LasR-Re	AAATCTAGATCAGAGAGTAAT AAGACC
lasB-promoter-For	AAAAGATCTGGTACCGCGCGT CCCGGAGCTGGGGG	lasB-promoter-Re	AAATCTAGATCAGAGAGTAAT AAGACC
LasR-LBD-For	AAAGAATTCTATGGCCTTGGTT GACGGTTTT	LasR-LBD-Re	AAAAAGCTTTCAGAGAGTAAT AAGACCCAA
qPCR-lasR-For	CTGTGGATGCTCAAGGACTAC	qPCR-lasR-Re	AACTGGTCTTGCCGATGG
qPCR-lasA-For	CCGTTCCTCTTCGTCTTGCT	qPCR-lasA-Re	AGTAACTCCGCCGAATAGCG
qPCR-lasB-For	ATCGACGTGTCCAAACTCCC	qPCR-lasB-Re	CCTTGACTTCGGTGATGGCT
qPCR-rhlR-For	GCCAGCGTCTTGTTCGG	qPCR-rhlR-Re	CGGTCTGCCTGAGCCATC
qPCR-pqsR-For	GCTTCGCCTGATCCCTTACA	qPCR-pqsR-Re	CTCACCGTATCGCAGAACGA
qPCR-phzM-For	ATTCGAATCTTGCTGCTGCG	qPCR-phzM-Re	GGAAGATCTCGAAGGCCACC
qPCR-phzS-For	CTTCGCAAGCAATCCTCGAC	qPCR-phzS-Re	CTTCTTCGTATTCGCGCAGG
qPCR-rhlI-For	AACCGAAAACCTGGGCTTCA	qPCR-rhlI-Re	TCACACCGCCATCGACAG
qPCR-16S-For	CGTCCGGAAACGGCCGCT	qPCR-16S-Re	CTCTCAGACCAGTTACGG
